# *In vitro* activity of N-acetylcysteine against *Stenotrophomonas maltophilia* and *Burkholderia cepacia* complex grown in planktonic phase and biofilm

**DOI:** 10.1371/journal.pone.0203941

**Published:** 2018-10-01

**Authors:** Simona Pollini, Vincenzo Di Pilato, Giulia Landini, Tiziana Di Maggio, Antonio Cannatelli, Samantha Sottotetti, Lisa Cariani, Stefano Aliberti, Francesco Blasi, Francesco Sergio, Gian Maria Rossolini, Lucia Pallecchi

**Affiliations:** 1 Department of Experimental and Clinical Medicine, University of Florence, Florence, Italy; 2 Department of Medical Biotechnologies, University of Siena, Siena, Italy; 3 Cystic Fibrosis Microbiology Laboratory, IRCCS Fondazione Cà Granda, Ospedale Maggiore Policlinico, Milan, Italy; 4 Department of Pathophysiology and Transplantation, University of Milan, Milan, Italy; 5 Internal Medicine Department, Respiratory Unit and Regional Adult Cystic Fibrosis Center, IRCCS Fondazione Cà Granda Ospedale Maggiore Policlinico, Milan, Italy; 6 Global Respiratory Medical Affairs, Zambon S.p.A., Bresso, Italy; 7 Clinical Microbiology and Virology Unit, Florence Careggi University Hospital, Florence, Italy; ENEA, Italian Agency for New Technologies, Energy and Sustainable Economic Development, ITALY

## Abstract

*Stenotrophomonas maltophilia* and *Burkholderia cepacia* complex (Bcc) have been increasingly recognized as relevant pathogens in hospitalized, immunocompromised and cystic fibrosis (CF) patients. As a result of complex mechanisms, including biofilm formation and multidrug resistance phenotype, *S*. *maltophilia* and Bcc respiratory infections are often refractory to therapy, and have been associated with a worse outcome in CF patients. Here we demonstrate for the first time that N-acetylcysteine (NAC), a mucolytic agent with antioxidant and anti-inflammatory properties, may exhibit antimicrobial and antibiofilm activity against these pathogens.

The antimicrobial and antibiofilm activity of high NAC concentrations, potentially achievable by topical administration, was tested against a collection of *S*. *maltophilia* (n = 19) and Bcc (n = 19) strains, including strains from CF patients with acquired resistance traits. Minimum Inhibitory Concentrations (MICs) and Minimum Bactericidal Concentrations (MBCs) ranged from 16 to 32 mg/ml and from 32 to >32 mg/ml, respectively. Sub-MIC concentrations (i.e., 0.25 × MIC) slowed down the growth kinetics of most strains. In time-kill assays, 2-day-old biofilms were more affected than planktonic cultures, suggesting a specific antibiofilm activity of NAC against these pathogens. Indeed, a dose- and time-dependent antibiofilm activity of NAC against most of the *S*. *maltophilia* and Bcc strains tested was observed, with a sizable antibiofilm activity observed also at 0.5 and 1 × MIC NAC concentrations. Furthermore, at those concentrations, NAC was also shown to significantly inhibit biofilm formation with the great majority of tested strains.

## Introduction

N-acetylcysteine (NAC) has long been used in clinical practice for its mucolytic, antioxidant and anti-inflammatory properties [1]. In addition, *in vitro* studies have revealed that NAC may exhibit some intrinsic antimicrobial and antibiofilm activity against several clinically relevant pathogens (including Gram-positive and Gram-negative bacteria and yeasts), although knowledge on this topic remains limited and the underlying mechanisms are poorly understood ([2] and references therein, [3–7]). The concentrations at which the antimicrobial and antibiofilm activities of NAC have been observed were variable but usually higher than those achievable by systemic routes of administration (i.e., oral, intramuscular or intravenous), which can result in peak plasma concentrations of 0.2–1.2 mg/ml [8]. However, NAC can also be administered topically, either by nebulization or direct instillation [2,9], and reach at the site of infection the higher concentrations needed for the antimicrobial and antibiofilm activity. Despite initial concerns about the potential negative interaction of NAC on antibiotic activity [10], two recent articles have demonstrated that NAC does not negatively affect the activity of the major antibiotic classes, with the exception of carbapenems [11,12].

By virtue of its multiple beneficial effects and high tolerability, a renewed interest in the potential therapeutic efficacy of topical NAC has recently emerged, especially for the management of cystic fibrosis (CF) and other chronic respiratory diseases (e.g. chronic obstructive pulmonary disease, and non-CF bronchiectasis) [2].

With regard to the difficult-to-treat pathogens associated with these diseases, NAC was previously shown to exert some antimicrobial and antibiofilm activity against *Pseudomonas aeruginosa* [2,13], but its activity against *Stenotrophomonas maltophilia* and *Burkholderia cepacia* complex (Bcc) remains unexplored.

*S*. *maltophilia* and Bcc are ubiquitous environmental microorganisms that act as relevant opportunistic pathogens in immunocompromised and hospitalized patients (especially patients in high-risk wards, such as Intensive Care Units), and those affected by CF [14–21]. Respiratory infections by *S*. *maltophilia* and Bcc are often recalcitrant to antibiotic therapy, as a consequence of complex and still largely unexplored mechanisms, which involve also a wide range of intrinsic and acquired antimicrobial resistance mechanisms, and the propensity to grow as biofilms [22,23].

Despite the pathogenic role of *S*. *maltophilia* in CF individuals has long been a matter of debate, chronic lung colonization by this pathogen has been recently associated with an increased risk of pulmonary exacerbation, lung transplantation and death [14,19,23].

Bcc is a versatile group of 21 species, of which *Burkholderia cenocepacia* and *Burkholderia multivorans* show a higher prevalence in CF infections compared to other Bcc species [24]. *B*. *cenocepacia* has a well-recognised impact on post-transplant morbidity and mortality, representing a contraindication to lung transplantation [25].

In order to find new drugs and combinations to improve the outcome of chronic lung colonization by *S*. *maltophilia* and Bcc in CF patients, a renewed interest has been recently focused on inhaled route of administration, which allow to achieve higher drug concentrations in the lungs, whilst limiting systemic toxicity [16,17].

Here we demonstrate, for the first time, that NAC may exhibit antimicrobial activity against *S*. *maltophilia* and Bcc grown either in planktonic phase or in biofilms, at concentrations achievable by topical administration.

## Materials and methods

### Bacterial strains tested, identification and susceptibility testing

A total of 38 strains were investigated (*S*. *maltophilia*, n = 19; Bcc, n = 19), including CF isolates (Table 1). Identification was performed by MALDI-TOF MS, and Bcc strains were also identified by PCR/sequencing of the *recA* gene [26]. In addition, species identification and Multi Locus Sequence Typing (MLST) of the six Bcc strains selected for time-kill assays and biofilm experiments were further determined following whole genome sequencing (WGS). Bacterial DNA was extracted using the phenol:chloroform method [27], and it was subjected to WGS with a MiSeq platform (Illumina, Inc., San Diego, CA), using a 2 × 300 bp paired-end approach, and reads were assembled using SPAdes [28]. Draft genome assemblies were used for downstream analyses at the Oxford PubMLST site (https://pubmlst.org/) and at the Center for Genomic Epidemiology site (https://cge.cbs.dtu.dk/services/KmerFinder/). The same approach was also used for three of the six *S*. *maltophilia* strains selected for time-kill assays and biofilm experiments. New ST-types were identified for *S*. *maltophilia* (i.e., Z120, ST334; Z155, ST335) and Bcc (i.e., Z136, ST1396; Z138, ST1398). Antimicrobial susceptibility was determined using the reference broth microdilution method [29].

**Table 1:** Features of the 38 *S*. *maltophilia* and *B*. *cepacia* complex strains investigated.

Strain^a^	Species		Origin^b^	Antibiotics^c^					N-acetylcysteine	
		MLST		MIC (μg/ml)					MIC (mg/ml)	MBC (mg/ml)
				CAZ	MEM	LVX	SXT	MIN		
Z63	*S*. *maltophilia*	-	BSI	2	-	≤0.25	0.5	0.125	16	32
Z64	*S*. *maltophilia*	-	BSI	64	-	4	2	2	16	>32
Z65	*S*. *maltophilia*	-	IAI	64	-	2	1	1	32	>32
Z66	*S*. *maltophilia*	-	LRTI	≤1	-	1	0,5	0.25	16	32
Z116	*S*. *maltophilia*	-	LRTI	16	-	2	0.5	0.25	32	>32
Z117	*S*. *maltophilia*	-	LRTI	64	-	0.5	0.5	0.25	16	>32
Z118	*S*. *maltophilia*	ST162	LRTI	8	-	2	0.5	0.25	16	>32
Z119	*S*. *maltophilia*	-	LRTI	32	-	2	0.5	0.50	32	>32
Z120	*S*. *maltophilia*	ST334	LRTI	32	-	1	0.5	0.5	16	32
Z128	*S*. *maltophilia*	-	LRTI	4	-	1	≤0.25	0.25	16	>32
Z129	*S*. *maltophilia*	-	LRTI	4	-	1	≤0.25	0.25	16	>32
Z130	*S*. *maltophilia*	-	IAI	16	-	16	0.5	2	16	>32
Z131	*S*. *maltophilia*	-	BSI	64	-	32	>8	1	16	>32
Z132	*S*. *maltophilia*	-	LRTI	2	-	16	1	1	32	>32
Z133	*S*. *maltophilia*	-	LRTI	2	-	1	1	0.25	32	>32
Z155	*S*. *maltophilia*	ST335	CF	32	-	4	>8	2	16	>32
Z156	*S*. *maltophilia*	-	CF	16	-	2	1	0.25	32	32
Z157	*S*. *maltophilia*	-	CF	4	-	2	0.5	1	32	>32
Z158	*S*. *maltophilia*	-	CF	16	-	0.5	≤0.25	0.25	16	>32
Z136	*B*. *multivorans*	ST1396	CF	>64	8	64	4	8	32	>32
Z161	*B*. *multivorans*	-	CF	>128	8	256	4	16	16	>32
LMG 16656	*B*. *cenocepacia*	ST28	CF	128	32	8	>8	16	16	>32
Z135	*B*. *cenocepacia*	-	CF	64	8	>256	4	64	32	>32
Z139	*B*. *cenocepacia*	-	CF	8	4	4	1	16	32	>32
Z140	*B*. *cenocepacia*	-	CF	16	16	>256	> 8	8	16	>32
Z142	*B*. *cenocepacia*	ST32	CF	2	4	32	8	8	16	>32
Z144	*B*. *cenocepacia*	-	CF	4	8	32	8	8	16	>32
Z146	*B*. *cenocepacia*	-	LRTI	16	16	128	8	4	32	>32
Z151	*B*. *cenocepacia*	-	LRTI	4	4	2	0.5	2	32	>32
Z160	*B*. *cenocepacia*	-	CF	16	16	32	8	4	16	>32
Z163	*B*. *cenocepacia*	-	CF	>128	16	128	1	8	16	>32
Z141	*B*. *cepacia*	-	CF	8	8	128	4	8	32	>32
Z145	*B*. *stabilis*	ST51	CF	128	8	32	8	4	16	>32
Z148	*B*. *stabilis*	ST51	LRTI	4	1	2	≤0.25	1	16	32
Z162	*B*. *stabilis*	-	CF	32	4	8	1	1	16	32
Z137	*B*. *metallica*	-	CF	4	4	16	2	1	32	>32
Z138	*B*. *seminalis*	ST1398	CF	2	2	64	2	2	16	>32
Z147	*B*. *contaminans*	-	LRTI	4	4	1	0.5	1	32	>32

^a^The 12 strains selected for planktonic time-kill assays and biofilm experiments are underlined.

^b^BSI, bloodstream infection; IAI, intra-abdominal infection; LRTI, lower respiratory tract infection; CF, cystic fibrosis.

^c^CAZ, ceftazidime; MEM, meropenem; LVX, levofloxacin; SXT, trimethoprim-sulfamethoxazole; MIN, minocycline.

### Preparation of NAC-containing medium

N-acetylcysteine stock solutions (100 g/L) were prepared immediately before use, by dissolving N-acetylcysteine powder (Zambon, Bresso, Italy) in sterile double-distilled water, pH adjustment at 6.5–6.8 with NaOH, and filtering through a 0.22-μm membrane filter. All experiments were performed in CAMHB (Becton Dickinson, Milan, Italy), starting from an appropriately concentrated medium in order to avoid broth dilution when testing high N-acetylcysteine concentrations.

### *In vitro* antimicrobial activity of NAC against planktonic cultures

Minimum Inhibitory Concentrations (MICs) and Minimum Bactericidal Concentrations (MBCs) of NAC were determined by broth microdilution (range of NAC concentration tested, 0.25–32 mg/ml) [29]. The effect of sub-MIC NAC concentrations (i.e., 0.25 × MIC) on the growth kinetics was determined in duplicate by recording the optical density at 600 nm over 20 h, using CAMHB inoculated with ~1.5 × 10^6^ CFU/ml.

Twelve strains (*S*. *maltophilia*, n = 6; Bcc, n = 6) (Table 1) were selected for time-kill assays with planktonic and biofilm cultures. The selected strains were representative of diverse origin (i.e., CF and non-CF LRTI), resistance phenotype (e.g., susceptibility to trimethoprim-sulfamethoxazole), and Bcc species. *B*. *contaminans* and *B*. *metallica* were not included at this stage, in order to test two *B*. *cenocepacia* belonging to diverse ST-types, and two *B*. *stabilis* strains belonging to the same ST-type, but showing diverse origin and resistance phenotype.

Planktonic time-kill assays were performed according to Clinical and Laboratory Standards Institute guidelines, in CAMHB [30]. Briefly, exponential phase bacterial cultures (OD_600_ ∼0.15) were diluted to ~5 × 10^5^ CFU/ml (final volume 10 ml) and exposed to 16 and 32 mg/ml NAC (i.e. 1 × MIC and 2 × MIC concentrations for the selected strains) over 24 h. Viable cell counts were determined by plating method after 3, 6 and 24 h of incubation (detection limit 25 CFU/ml). Data were obtained from at least two independent experiments, with two replicates per condition per experiment.

### *In vitro* antibiofilm activity of NAC

Biofilm susceptibility testing was performed using the Nunc-TSP lid system (Thermo Fisher Scientific, Waltham, MA, USA), as described previously [31]. Briefly, 2-day-old biofilms were challenged with daily refreshed NAC-containing medium (i.e., 8, 16, and 32 mg/ml) at 35°C under static conditions, and the effect of NAC was evaluated after 1 and 3 days of exposure. After the exposure time, biofilms were washed twice with 200 μl of phosphate-buffered saline (PBS) (Sigma Aldrich, Milan, Italy) to remove loosely adherent bacteria, and sessile cells were removed from pegs by sonication for 30 min (Elma Transsonic T 460, Singen, Germany) in 200 μl of tryptic soy broth (TSB) (Oxoid, Milan, Italy) supplemented with 0.1% Tween 20 (Sigma Aldrich) (i.e., the recovery medium). Mean viable cell count per peg (log CFU/peg) was then determined by plating 10 μl of appropriate dilutions of the recovery medium onto tryptic soy agar (TSA) (Oxoid) and incubating for 48 h at 35°C (detection limit, 1.3 log CFU/peg). Data were obtained from at least two independent experiments, with at least four replicates per condition per experiment.

The capability of NAC to affect biofilm formation was evaluated with biofilms grown for 72 hours in CAMHB in the presence of 0, 4, 8 and 16 mg/ml NAC concentrations in daily refreshed medium (at 35°C, static condition). After the incubation times, viable cells were counted as in eradication experiments (see above).

### Statistical analysis

Statistical analysis was performed using GraphPad Prism version 6.0 (San Diego, CA, USA). D’Agostino-Pearson, Shapiro-Wilk and Kolmogorov-Smirnov normality tests were used to test for Gaussian distribution. Concerning biofilm experiments, for each time point multiple comparison tests were applied to assess differences of biofilms exposed to diverse NAC concentrations compared to controls. One-Way ANOVA with Dunnett’s correction and Kruskal-Wallis test with Dunn’s correction were performed in case of Gaussian or not Gaussian distribution, respectively. Unpaired t-test with Welch’s correction was used for growth curves analysis.

## Results and discussion

### *In vitro* activity of NAC against *S*. *maltophilia* and Bcc grown in planktonic phase

MICs of NAC for the tested *S*. *maltophilia* and Bcc strains were 16 or 32 mg/ml, whereas MBCs were 32 mg/ml for four strains (*S*. *maltophilia*, n = 2; Bcc, n = 2), and >32 mg/ml for the remaining ones (Table 1).

**Fig 1:**
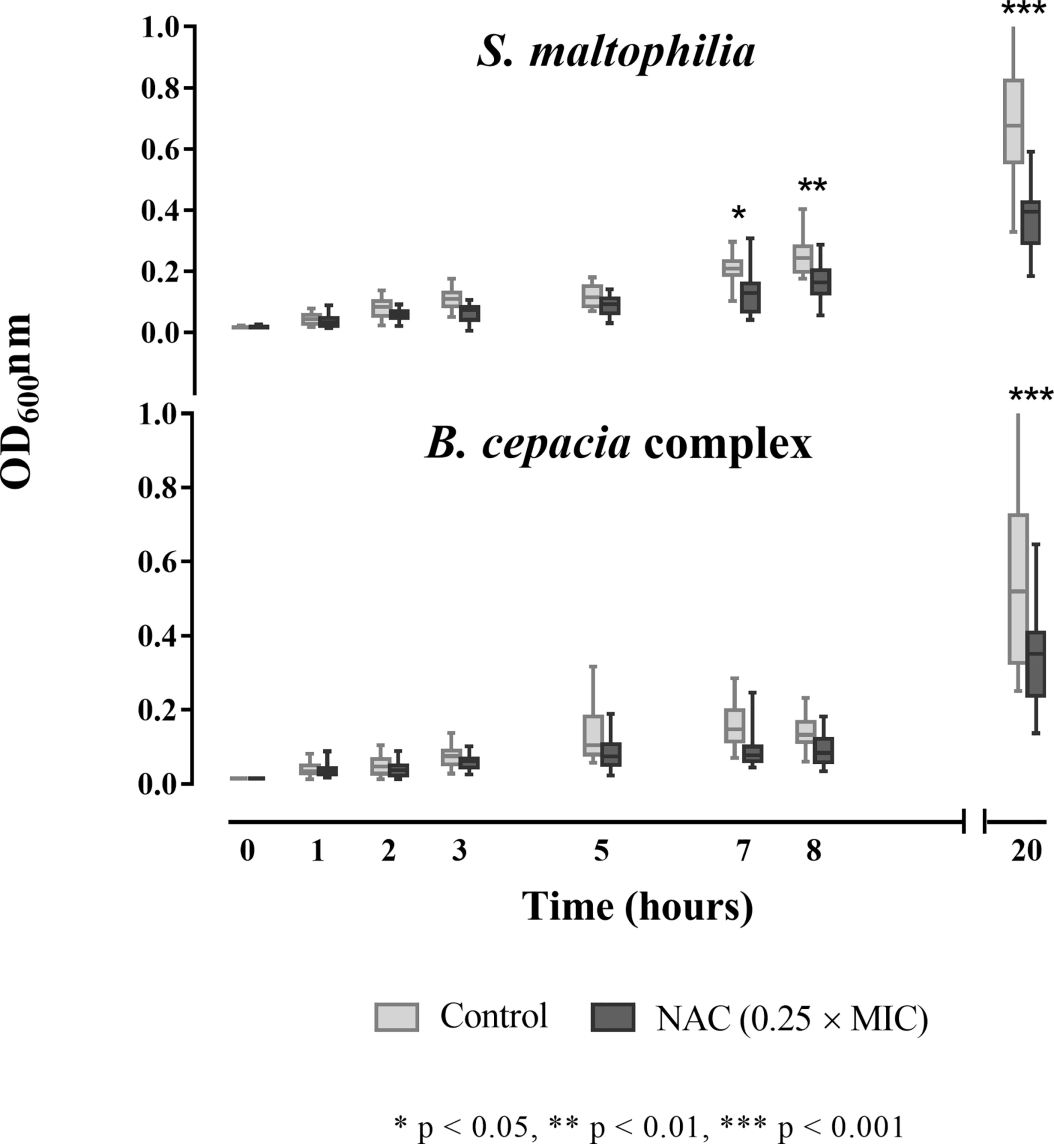
Boxplot representation of the effect of sub-MIC NAC concentrations on the growth kinetics of 19 *S*. *maltophilia* and 19 Bcc clinical isolates. Data from two independent experiments. Boxes indicate from the 25^th^ to the 75^th^ percentiles, and whiskers indicate the minimum and maximum values.

Sub-MIC NAC concentrations (i.e., 0.25 × MICs) were able to slow down the growth kinetics of most of the strains tested, with *S*. *maltophilia* being the most affected species (especially after 20 h of incubation) (Fig 1).

**Fig 2:**
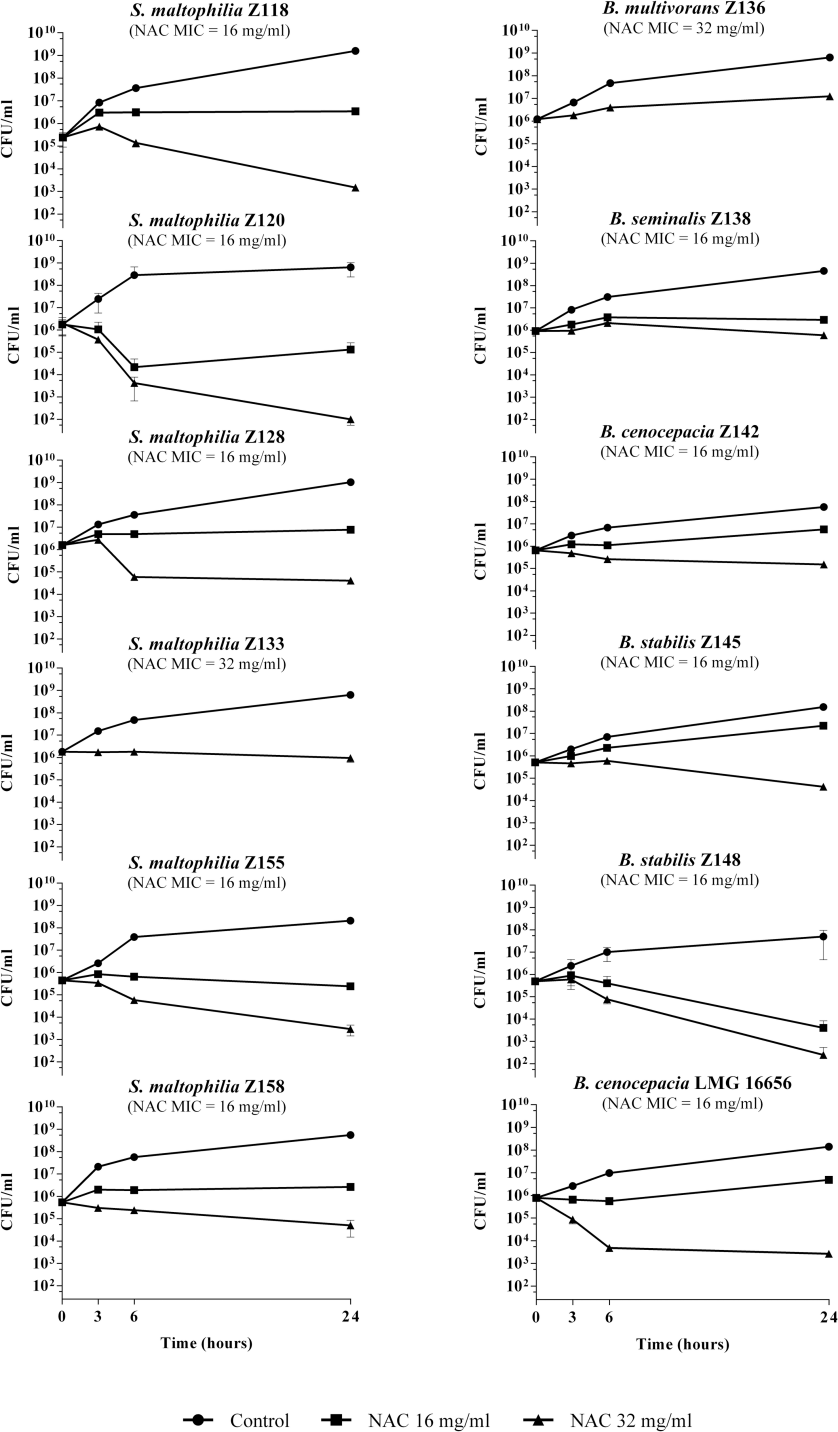
Time-kill assays of NAC for *S*. *maltophilia* and Bcc planktonic cultures. Data from at least two independent experiments, with two replicates per condition per experiment. Mean values with standard deviation are plotted. The *x* axis is set at the limit of detection (i.e., 25 CFU/ml).

In time-kill assays performed with planktonic cultures, NAC at 1 × MIC did not exert bacterial killing against the strains tested, except for *S*. *maltophilia* Z120 (i.e., 1.3 log CFU/ml reduction after 24 h) and *B*. *stabilis* Z148 (i.e., 2.1 log CFU/ml reduction after 24 h) (Fig 2). At 2 × MIC concentrations, NAC was bactericidal (i.e., reduction of ≥3 log of the initial bacterial inoculum) for these two strains (i.e., 4.2 and 3.2 log CFU/ml reduction for *S*. *maltophilia* Z120 and *B*. *stabilis* Z148, respectively), and reduced of at least 1 log CFU/ml the viable cell counts for four additional *S*. *maltophilia* and two Bcc strains (range, 1.1–2.5 log CFU/ml) (Fig 2).

### *In vitro* activity of NAC against *S*. *maltophilia* and Bcc grown in biofilms

**Fig 3:**
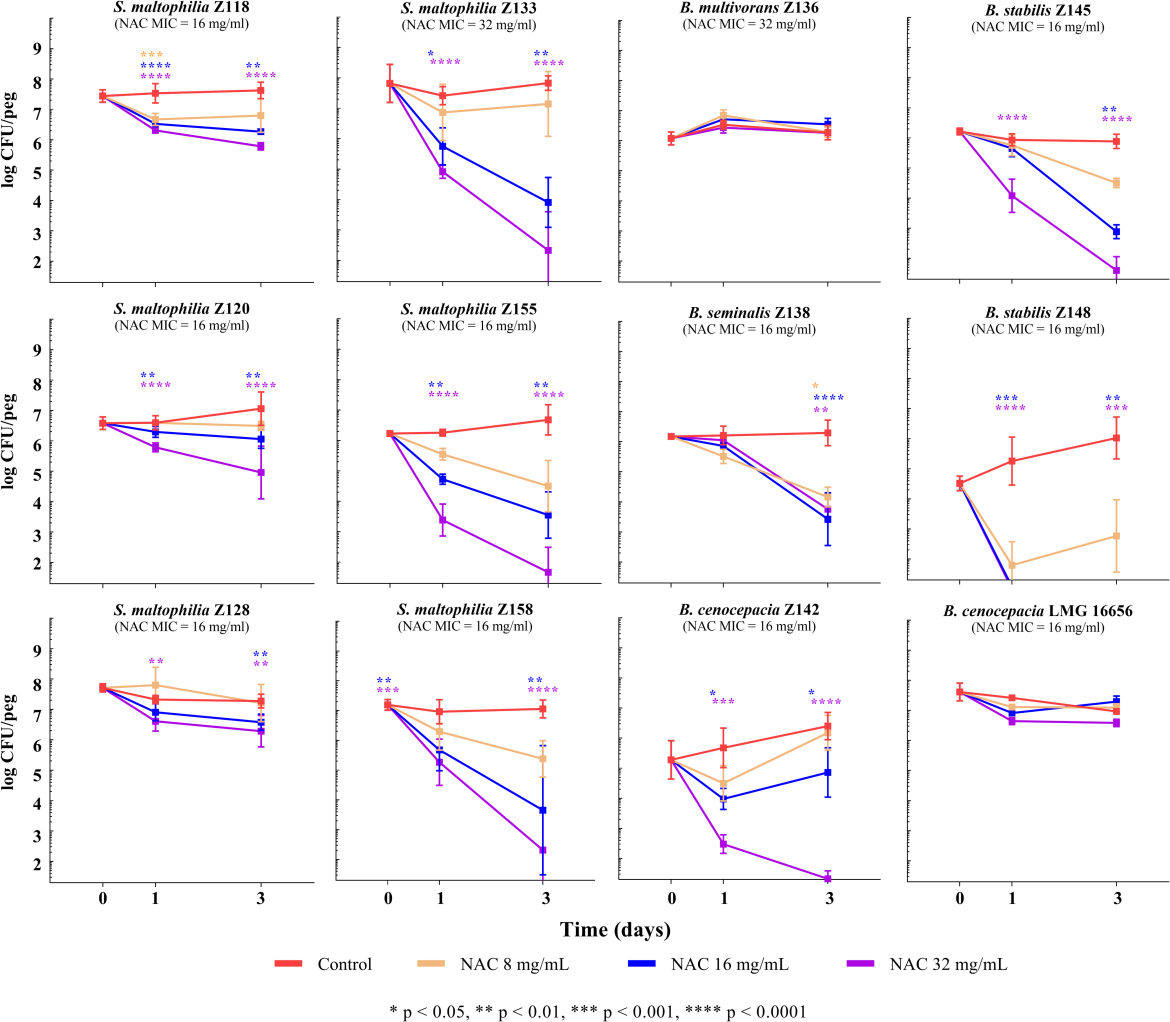
Time-kill curves of NAC for 2-day-old biofilms of *S*. *maltophilia* and Bcc. Data from at least two independent experiments, with at least four replicates per condition per experiment. Median values with standard deviation are plotted. The *x* axis is set at the limit of detection (i.e., 1.3 log CFU/peg).

Time-kill assays performed with 2-day-old biofilms (average, 7.16 ± 0.63 and 5.98 ± 1.04 log CFU/peg for *S*. *maltophilia* and Bcc, respectively) revealed a dose- and time-dependent antibiofilm activity of NAC against most of the *S*. *maltophilia* and Bcc strains tested (except for *B*. *multivorans* Z136 and *B*. *cenocepacia* LMG 16656) (Fig 3). Interestingly, with six strains, including three *S*. *maltophilia* and three Bcc, a sizable antibiofilm activity was already observed at 0.5 × MIC and 1 × MIC NAC concentrations, which were found to determine a reduction of viable cells of ≥1 log CFU/peg and ≥2 log CFU/peg after a 3-days exposure, respectively (Fig 3). Considering the substantial lack of killing activity of 1 × MIC NAC concentrations against planktonic cultures of the same strains (Fig 2), these data would point towards a specific antibiofilm activity of NAC against these pathogens. Furthermore, at the highest concentration tested (i.e., 32 mg/ml), NAC exerted a bactericidal effect (i.e., reduction of ≥3 log CFU/peg) against half of the strains grown in biofilms (Fig 3).

**Fig 4:**
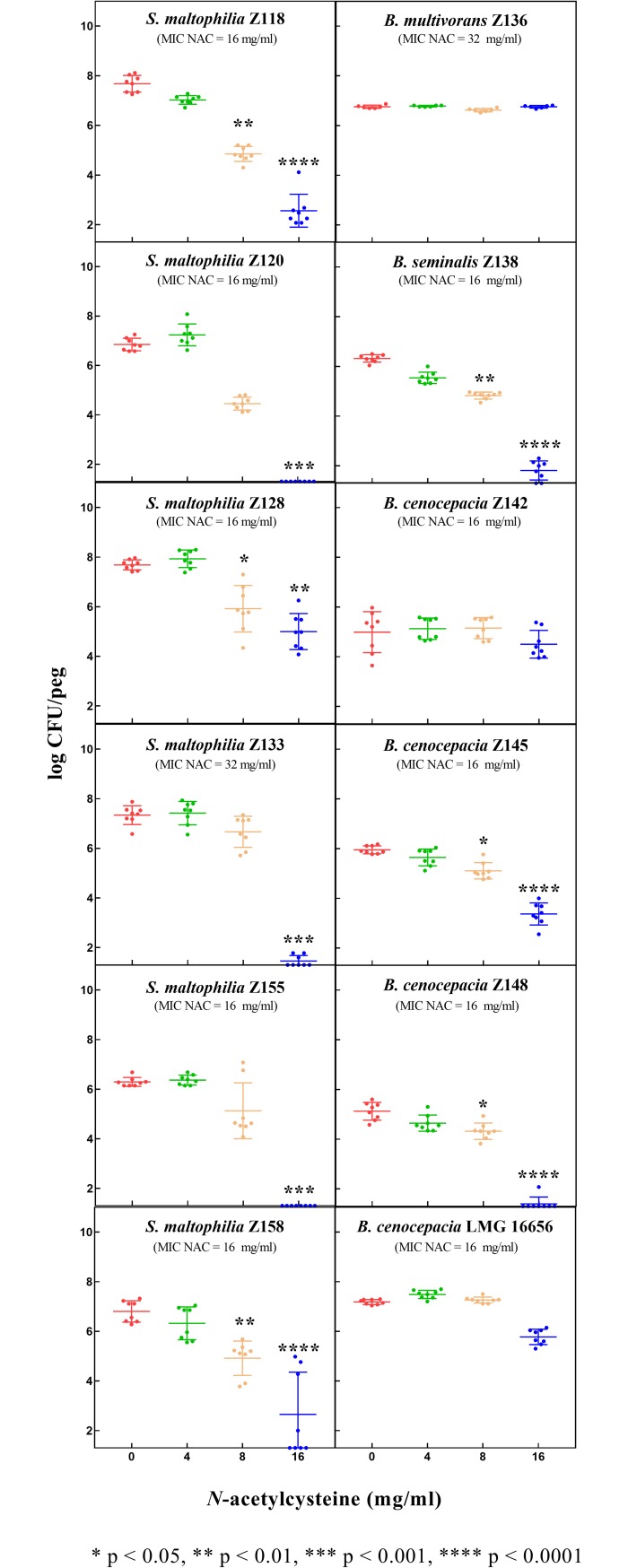
Effect of NAC on *S*. *maltophilia* and Bcc biofilm formation (72-hours growth). Data from at least two independent experiments, with at least four replicates per condition per experiment. Median values with standard deviation are plotted. The *x* axis is set at the limit of detection (i.e., 1.3 log CFU/peg).

Finally, NAC at 0.5 × MIC or 1 × MIC concentrations was also shown to significantly affect biofilm formation of the great majority of tested strains (i.e., all except *B*. *multivorans* Z136 and *B*. *cenocepacia* Z142) (Fig 4). Results were overall consistent with those obtained in biofilm eradication experiments, except for *B*. *cenocepacia* Z142, for which NAC had a relevant antibiofilm effect on preformed biofilms, while no effect in inhibiting biofilm formation was observed (Figs 3 and 4).

The diverse response to NAC exposure observed in biofilm experiments among strains of the same species, exhibiting similar NAC MIC, suggests a strain-dependent antibiofilm activity of NAC against these pathogens. The reasons accounting for this phenomenon are difficult to hypothesize, since mechanisms underlying the antibiofilm activity of NAC remain still largely unknown. In addition, the different results obtained with *B*. *cenocepacia* Z142 in biofilm prevention and eradication experiments further suggest a complex and multifactorial antibiofilm activity of NAC.

## Conclusions

The results of this study demonstrated for the first time that high NAC concentrations, achievable by topical administration (inhalation or direct instillation), may exert a relevant antimicrobial and antibiofilm activity against *S*. *maltophilia* and Bcc, including CF isolates with acquired resistance traits. These difficult-to-treat pathogens have been increasingly recognized as relevant pathogens in hospitalized, immunocompromised and CF patients, being associated with a worse outcome in CF patients [14–21]. Interestingly, the antibiofilm activity appeared to be only partially related to the antimicrobial activity, suggesting that NAC might act by inducing biofilm disgregation or be more active against biofilm-associated cells than planktonic cells. Further studies are needed to understand the mechanisms of such a phenomenon, considering that the antibiofilm properties of NAC have been hypothesized to be multifactorial (e.g. perturbation of cell physiology, direct interaction with crucial components of the matrix) [2], and have not been fully elucidated so far.

Although the low number of strains tested did not allow to speculate on potential associations between Bcc species and NAC susceptibility, the differences observed with the two *B*. *cenocepacia* strains (*B*. *cenocepacia* Z142 and LMG 16656) would rather suggest a strain-specific susceptibility. Based on the present findings, further studies aimed at expanding the number of strains and Bcc species tested and addressing the potential antibiofilm synergism of NAC plus conventional antibiotics are strongly encouraged.
